# Perceptions Toward the Use of Digital Technology for Enhancing Family Planning Services: Focus Group Discussion With Beneficiaries and Key Informative Interview With Midwives

**DOI:** 10.2196/25947

**Published:** 2021-07-28

**Authors:** Hind Yousef, Nihaya Al-Sheyab, Mohannad Al Nsour, Yousef Khader, Malika Al Kattan, Marco Bardus, Mohammad Alyahya, Hana Taha, Mirwais Amiri

**Affiliations:** 1 Global Health Development | Eastern Mediterranean Public Health Network Amman Jordan; 2 Department of Allied Medical Sciences Faculty of Applied Medical Sciences Jordan University of Science and Technology Irbid Jordan; 3 Department of Community Medicine, Public Health and Family Medicine Faculty of Medicine Jordan University of Science & Technology Irbid Jordan; 4 Department of Health Promotion & Community Health American University of Beirut Beirut Lebanon; 5 Department of Health Management and Policy Jordan University of Science and Technology Irbid Jordan

**Keywords:** family planning, mobile apps, social media, digital technology, contraceptives

## Abstract

**Background:**

Modern family planning (FP) methods allow married couples to discuss and determine the number of children and years of spacing between them. Despite many significant improvements in FP services in Jordan, there are still many issues related to the uptake of FP services for both host communities and Syrian refugees, due to limitations in the health care system based on public health facilities. Digital technologies can provide opportunities to address the challenges faced in the health system, thus offering the potential to improve both coverage and quality of FP services and practices.

**Objective:**

The aim of this study was to explore the perceptions of Jordanian women, Syrian refugees, and midwives in Jordan toward the use of digital health technology to support and enhance access to FP services.

**Methods:**

We employed a qualitative study based on semistructured, face-to face key informative interviews with 17 midwives (providers) and focus group discussions with 32 married women of reproductive age (clients). Both midwives and clients were recruited from 9 health centers in 2 major governorates in Jordan (Irbid and Mafraq), where 17 in-depth interviews were conducted with midwives and 4 focus groups were conducted with the women. Each focus group included 4 Syrian refugees and 4 Jordanian women. The transcribed narratives were analyzed using inductive thematic analysis.

**Results:**

Three major themes were derived from the narratives analysis, which covered the pros of using digital technology, concerns about digital technology use, and the ideal app or website characteristics. Ten subthemes emerged from these 3 main themes. Overall, midwives and women (Syrian refugees and host communities) agreed that digital technology can be feasible, cost-effective, well accepted, and potentially beneficial in increasing woman’s awareness and knowledge regarding the FP methods and their side effect. Furthermore, digital technology can assist in enabling women’s empowerment, which will allow them to make better decisions regarding FP use. No harmful risks or consequences were perceived to be associated with using digital technology. However, several concerns regarding digital technology use were related to eHealth literacy and the accuracy of the information provided. Midwives were mainly concerned about the patients who would rely mostly on the technology and choose to avoid consulting a health care professional.

**Conclusions:**

As perceived by midwives and women, incorporating digital technology in FP services can be feasible, cost-effective, well accepted, and potentially beneficial in increasing woman’s awareness regarding the FP methods and their side effect. It may also empower the women to play an active role in the shared (with their husband and family) decision-making process. Therefore, digital technologies are recommended to address the challenges faced in health system and to improve both the coverage and the quality of FP services and practices.

## Introduction

### Family Planning Services in Jordan

Family planning (FP) is the necessary manifestation of the basic human rights where married couples get to determine the number of children and years of spacing between them [1,2]. FP is widely used throughout the world, but the uptake of the individual methods varies enormously between different countries and is relatively low in low- and middle-income countries (LMICs). In a recent scoping review, the unmet need for FP was reported to range from 20% to 58% across 34 studies conducted in LMICs [3]. According to the World Health Organization (WHO), FP uptake is generally low in some specific segments of the population, such as refugees, women in the postpartum period, adolescents, migrants, and urban slum dwellers [4]. The United Nations High Commissioner for Refugees (UNHCR) conducted a multicountry assessment on FP services offered to refugees in Bangladesh, Djibouti, Jordan, Kenya, Malaysia, and Uganda [5]. The assessment showed that 15-19-year-old married refugee women had higher unmet need, were less likely to use contraception, and had lower awareness compared with older women aged 20-49 years.

In Jordan, while a 2016 study found that 42% of married Syrian refugees never used modern FP methods [6], the latest population and family health survey according to the Demographic and Health Surveys program showed that the unmet need for contraception among Syrian refugees was 19%, compared with 14% among Jordanian women [7]. Jordan’s fertility rate showed only a minor decline from 3.7% in 2002 to 3.5% in 2012 and a further decline to 2.7% in 2017 [1]. This drop was significant considering the noticeably high birth rates among Syrian refugees, which could have indirectly increased the overall Jordanian fertility rates in the last decade [2,4]. The main factor attributed to the reduction in fertility rate in Jordan was the adoption of FP strategies among Jordanian families [8].

Accessing FP and health services is difficult in countries of the Eastern Mediterranean, such as Jordan and Lebanon, whose health care systems had to sustain the influx of large numbers of Syrian refugees. According to the UNHCR, as of June 2019, the number of registered Syrian refugees exceeds 664,000 in Jordan [9]. In this country, the Ministry of Health provides free primary health care, including maternal and child health, to all residents including Syrian refugees. Yet, the use of any FP method has fallen from 61% to 52% between 2012 and 2017 with more reduction in traditional FP methods (from 23% to 11%) [7]. Surprisingly, more than half of Jordanian women of childbearing age reported not using any type of modern FP methods [3], a fact that highlights the gap between the reality and the Sustainable Development Goals’ intention of improving the use of modern FP methods to 80% [4]. Further, despite the significant improvement in FP services through providing free FP methods and counseling to both host communities and Syrian refugees, there are many issues that hamper the access to FP services due to the manner in which public health facilities operate. For instance, failure to adapt to the latest technology is one of those issues, as it has not been able to bridge the gender role gap between women and men culturally.

### Barriers to Accessing Family Planning Services

According to a recent UNHCR report [5], barriers to FP use among refugees globally include availability, accessibility, and acceptability of FP services. Acceptability barriers include, for example, religious opposition, perceived social pressure from family members, and language barriers in dealing with local health care providers; availability of services and accessibility barriers include, for example, distance from health care facilities and costs of transport and of a doctor’s consultation [5]. These factors include also discrimination and other biases from the providers, which impact the quality of care, or perceived social pressure related to large expected family sizes from spouses, parents, and relatives [10]. Among Jordanian and Syrian women, available research shows that common barriers to FP use include fear of adverse side effects and complications [11], which leads to uptake of less effective traditional methods (eg, withdrawal and condoms). This was coupled with limited awareness about fertility limitation and FP in general [12,13], and limited sexual health knowledge [14]. From a sociocultural perspective, most married couples, being both Jordanian and Syrian, believe that Islam promotes birth spacing while encouraging having a large family size. As a matter of fact, the Arab culture appears to be a significant determinant of family size, having preference for large families and male children, facts that can have a significant influence on FP decisions [15].

### The Potential of Digital Technologies for Family Planning

Using digital technologies for health promotion has become a prominent practice for utilizing both routine and innovative information and communications technology in addressing health needs [16]. Various reviews focusing on sexual and reproductive health interventions showed that mobile technologies can improve the uptake of services [17,18]. For example, SMS text messages can be used as reminders to improve attendance to doctors’ appointments and compliance with medications [19-21]. In LMICs, some studies have tested the use of mobile SMS text messages to reduce unwanted pregnancy among clients [22], or the usage of mobile-delivered health communication campaigns to encourage discussion about FP [23]. Digital health interventions were also used to provide FP counseling with a mobile job aid [24,25] and training for health care workers using SMS text messages and interactive voice response [26]. However, limited evidence exists on the use of these technologies in the Arab world and among refugees [27,28] and for the purpose of promoting FP services.

According to the Global Digital Health Index (GDHI) [29], Jordan has a fully functional governance structure that monitors the implementation of digital health strategies. The workforce lacks some training in digital health and thus requires preservice training. Similarly, digital technologies can provide opportunities to address the challenges faced in the health system, thus offering the potential to improve both the coverage and quality of health services and practices [16]. Some research has shown that women are increasingly interested in contraceptive tools utilizing mobile technology, and most women expect them to be science based [30,31]. The majority of mobile apps available support natural FP methods, which are recognized as the least effective FP method [32]. However, the implementation of digital health technologies has been extensively used only in the short term and without rigorous examination of benefits and harms on the health system and people’s quality of life [33]. Moreover, health care providers and stakeholders need to understand what motivates and hinders people to use digital health interventions [32]. While some qualitative studies have explored the perceptions toward FP among community members and health providers in LMICs or Global South countries such as South Africa [33], to the best of our knowledge, no previous studies have been conducted in Jordan to explore the perceptions of the midwives and clients (Jordanian women and Syrian refugees) toward digital technology use for promoting FP [31].

### Objectives

This study aimed to explore the perceptions of Jordanian women, Syrian refugees, and midwives in Jordan toward the use of digital health technology to support access to FP services. This study constitutes the formative research activity to develop a digital solution to increase the uptake of FP services among Syrian refugees and host communities in Jordan.

## Methods

### Study Design

#### Overview

A qualitative research was conducted among 2 study populations: Jordanian women and Syrian refugees (the clients), and midwives who provide FP services (the providers). We conducted focus group discussions (FGDs) with clients and one-to-one, face-to-face in-depth interviews with the providers. The objective of the focus groups and interviews was to explore the participants’ perceptions about using digital technology (such as mobile apps, SMS text messages, and other tools) as a means to receive counseling and obtain information on FP.

#### FGDs With Clients

Data were collected between January and March 2020. The research team recruited a purposive sample (n=32) of married women of reproductive age through the midwives working at the International Rescue Committee (IRC) and Comprehensive Health Centers in 2 governorates in northern Jordan. Four FGDs were conducted among women attending the IRC clinics and Ministry of Health primary care facilities. Each FGD included 4 Jordanian and 4 Syrian women. To obtain a broader view of the participants’ perceptions about FP, women of different ages and different levels of education were invited. It is important to consider that it was initially planned to conduct 15 FGDs with clients. However, after conducting 4 FGDs, the research team realized saturation was reached and sufficient information was obtained.

The research team developed an FGD interview guide. A moderator guided the discussion and encouraged all women to share their perception and to express their own views. Comprehension probes were used if needed to clarify responses. The focus groups lasted from 90 to 120 minutes.

#### Interviews With Providers

We conducted in-depth interviews with all the midwives (n=17) who provide FP services in 9 different health centers in 2 major governorates in Jordan: Irbid and Mafraq. Two of the 9 health centers primarily provide services for Syrian women. All the midwives were previously trained to provide FP counseling and services. Data were collected during January-March 2020. Two trained female investigators (HY and NA-S) conducted the individual interviews with the midwives using an interview guide designed by the research team. This guide covered the major concepts to be discussed during the interviews. Other more specific questions and probes were also asked, as appropriate, but were not initially included in the interview guide but emerged during the active discussion. All interviews were held in a quiet setting at a convenient place to all midwives after obtaining their approval and consent. Researchers conducted the interviews in the local Arabic dialect. Interviews lasted for around 30-45 minutes depending on the midwives’ level of interaction and sharing experiences.

A digital voice recorder was used for the FGDs and the face-to-face interviews, as it allowed easy management of interviews and it recorded high-quality audio, which facilitated the transcription.

### Ethical Approval

The study received ethical approval from the Research Ethics Committee of the Jordan University of Science and Technology and the Ministry of Health in Jordan (Ref.: 6/127/2019, received on September 12, 2019). During participant recruitment and before data collection, study participants were given verbal and written information about the study aims and objectives. Furthermore, they were informed that their participation in this study was confidential and voluntary, and that they could withdraw from the study at any given point without negative consequences to them. All participants signed a consent form, which included the consent to audiotape the interviews and FGDs.

### Data Analysis

All discussions were transcribed verbatim. Data were analyzed using inductive thematic analysis [34,35]. Preliminary analysis was conducted after each interview to get a general impression of the results, which allowed for early identification of areas that needed additional clarifications from the study participants. Researchers conducted the thematic analysis in its original language (Arabic) to maintain trustworthiness and credibility of the findings, which could have been lost by initial, inaccurate translation [36]. Translation into English was commenced after themes were generated and for extraction of quotations. The initial phase of the thematic analysis, as described by Braun and Clarke [34] “becoming familiar with the data,” was initiated by reading and re-reading the transcripts several times. Repeated reading contributed to get a better understanding and enhanced researchers’ familiarity with the data. Following the initial stage, *codes were generated* from the data set. Coding was carried out by systematically organizing and gaining meaningful characteristics of data related to the research question. One of the researchers (HA) began the process of initial coding; all transcriptions were coded one by one. This process was repeated until coding consensus was reached. For the transcribed data from FGDs, data analysis was undertaken manually through coding and generating categories and themes.

Concerning the transcripts for the face-to-face interviews with midwives, Qualitative Data Analysis in R (RQDA) was used for analysis. The third phase “searching for themes” focused on a broader level of analysis and involved the researcher identifying suitable themes to which codes could be attributed [34,35]. Initial codes pertinent to research question were integrated into themes considering how relationships were formed between codes and potential themes. To visualize and explore trends and relationships in the source data, codes, and themes, a tree mapping was formulated using RQDA. Derived themes were reviewed in phase four of the analysis through a cyclical process that involves back-and-forth movements between phases of data analysis until a consensus was reached on the final themes. Consequently, in phase five, “Defining and naming themes” was completed, through refining existing themes and subthemes that will be presented in the final analysis [34,35].

## Results

### Study Participants

Four FGDs were conducted among 32 women of reproductive age attending IRC clinics and Ministry of Health primary care facilities. In addition, 17 midwives aged between 27 and 57 years and with 1 month to 25 years of experience were interviewed. All midwives were previously trained to provide FP counseling and services.

### Themes and Subthemes Categorization

The data generated through the focus groups and interviews were organized into 3 major themes, combining both the clients and providers’ perspectives: (1) advantages of digital technology for FP, (2) concerns about the use of digital technology for FP, and (3) the characteristics of an ideal app for delivering an intervention on FP methods. Within the themes, 10 subthemes were generated. The themes and subthemes are summarized in Textbox 1.

**Themes (bold) and subthemes of midwives and women’s perceptions about family planning in Jordan.**

**Advantages of digital technology for family planning**

- Raising awareness on family planning
- Convenience and cost efficiency
- Empowering women

**Concerns about the use of digital technology**

- Quality and accuracy of the information
- eHealth literacy
- Need for advice from a health care professional
- Internet access and health literacy

**Ideal app or website characteristics**

- Ease of understanding and visualizations
- Privacy concerns
- Free access

### Advantages of Digital Technology for Family Planning

Both clients and providers perceived digital technology as beneficial for FP for several reasons: it helps in raising awareness, is convenient and cost-effective, and may help in empowering women.

#### Raising Awareness on FP

Digital technology was perceived by the midwives and clients as an effective tool to increase the women and their husbands’ awareness about FP. It was perceived to be effective in increasing their access to information about the different FP methods and their side effects, which will allow them to make better decisions on FP use. A midwife explained*:*

If a woman used a method and suffered from a side effect that the midwife did not talk about before, she can open an application and learn more about the side effect.

A woman said, “It would be so helpful as I can take my time reading about each method.” Moreover, some midwives believe that women tend to absorb information better when they search for it personally and they find this information more convincing. Another benefit of digital technology is that it could inform them about other aspects such as nutrition, physical, and mental health related to FP*.* A woman said: “I can gain information about nutrition, physical, and mental health related to FP.”

Finally, digital technology was not only perceived as a tool to access information about FP for the patients, but midwives also perceived it as a source of new information for them. An interviewee explained “even if I’m a midwife, I download Daleel Hamly application (ie, my pregnancy guide) when I get pregnant, as I find some new information I’ve never studied before.”

#### Convenience and Cost Efficiency

According to both providers and clients, digital tools were perceived as a convenient and cost-efficient method to promote FP as it can help women access FP-related information and answers their questions at any time based on their availability and free time. One client explained, “It would be great to obtain medical consultations through the websites while I am home, not worrying about leaving my children home alone.”

Moreover, technology can help women to connect with health care providers without having to go to the health care center, especially for those who may not be able to seek FP services at the centers due to distance or the lack or inability to pay for transportation. A participant explained, “I live 30 minutes away from the health center and takes me time to find a taxi or bus and costs me at least 3 Jordanian dinars.”

According to both providers and clients, technology can help women and their husbands decide about their preferred FP method before visiting the health care center, which may save both time and effort. A midwife said, “on the contrary, it would be useful if they were previously educated at home and they have already made up their minds before coming to the center to get the method.” A woman explained, “My husband and I can reach mutual agreement and understanding of the preferred method we want to use before coming to the center to get the method as this will reduce consultation time.”

#### Empowering Women

Most of the clients (Syrian refugees and Jordanian women) and providers perceived that digital technology can empower women because its information help women to convince their husbands to be involved in FP counseling sessions, especially if the husband cannot accompany women to counseling. Midwives explained that women can try to convince their husbands and in-laws of a certain method, especially if the husband cannot accompany women to counseling or if the midwives are embarrassed to explain the use of certain methods to the husband, by showing them a full explanation of it through a mobile app or website, as people tend to believe the information they find online. Clients and midwives stated that technology such as videos of teaching communication skills can teach women how to communicate with their husbands to seek FP services.

Additionally, midwives perceive that technological developments can increase the educated women’s awareness about the available FP methods by enabling them to access a variety of topics easily and by giving them the chance to share and discuss their opinions and make their own decisions. Another reason was provided by Syrian and Jordanian women who mentioned that mobile technology can empower them through informing them of ways to take good care of their physical and mental health, how to space between pregnancies, and what general information each woman must know about the reproductive health.

### Concerns About the Use of Digital Technology

Despite the high level of acceptance and enthusiasm toward the use of technology for the purpose of FP, some study participants raised a number of concerns related to the quality and accuracy of information, the ability to use technologies, the need for advice from a health care professional, limited internet access, and limited health literacy.

#### Quality and Accuracy of the Information

Women clients had concerns that FP apps may not have sufficient or updated information. A participant said, “I need to make sure that the information listed is correct and up to date.” Another participant added, “There might be new types of FP methods or new types of pills and I don’t know about.” Furthermore, one of the biggest fears of some of the interviewed midwives is that the technological services may provide wrong information that is likely to scare women from seeking the FP methods (eg, rare side effects and impact of some methods on weight or infertility). In addition, some midwives were concerned that apps may not be developed in the right way, may not include sufficient information, or may not be monitored or supervised by experts. For example, they stated that the apps might include incorrect terminology or unclear sentences, especially those that are translated from one language to another, hence appearing untrustworthy.

#### eHealth Literacy

Both midwives and women raised the concern about sharing misinformation due to their inability to correctly understand and explain the concepts included in FP apps or websites. These problems can be linked to the eHealth literacy model [37], which entails 6 domains of literacy, including basic or functional literacy (ie, the ability to read a text); scientific, media, information (ie, the ability to understand scientific literature, how media work, and how information is shared and produced, respectively); health (ie, the ability to process and use health-related information on a specific topic to make decisions); and digital literacy (ie, the ability to use technology to seek information). eHealth literacy is closely linked to the level of education, which can be a barrier to interventions delivered through digital technology. Education plays a role especially among Syrian women, as, on average, their educational level is lower than that of Jordanian women. One Syrian participant stated, “usually in Syria, girls get off married early before they complete their 8^th^ grade,” so they drop-out from school at an early age, not completing their education. This, of course, means that some of these women will not be able to read and understand some textual information. For example, both clients and providers mentioned the problems related to functional literacy: one woman mentioned that “I am worried that I will not understand what is written”; several providers expressed concerns about the readability of the text included in the apps, noting that they had observed that women can speak well but are not able to read. Some other women mentioned problems of scientific and health literacy that were linked to the difficulty of understanding some medical terms. Additionally, both midwives and women expressed concerns about their ability to use technology, as not all clients have familiarity with apps and websites.

#### Need for Advice From a Health Care Professional

Some midwives were hesitant about the use of technology in the FP-related services, as one thinks that women tend to take advice from each other, even if they are wrong. Some midwives think that technology cannot replace face-to-face consultations by the midwives, as women trust the health care providers and their professional experience. As for the clients, some believe that they might not know which type of FP method is best for their body without consulting their midwives, as one participant said, “How can I tell if this type is good for me without consulting the midwife.”

In addition, 2 providers were concerned that digital technology apps would increase their workload by taking a longer time to counsel women. They believed women might have more questions about the many methods of FP after using apps, resulting in more time to counsel women on these additional methods.

Furthermore, it is important to know the medical history of each woman before giving her any advice regarding FP. A midwife working with Syrian women argued that technology may only provide a brief explanation of a certain medical condition, but not all of the smallest details and that it may not be able to warn the woman in case she needs an urgent medical intervention, including misunderstanding and information overlap, which may lead to making wrong decisions on FP. For example, a midwife working with Syrian women said, “Women may associate some of the side-effects presented through the technological services to the family planning method they use, even if the complications may be caused by other medical problems.”

When women were asked about risks and consequences that might be associated with the use of digital technology, specifically about gender-based violence, they all stated that no harmful risks or consequences are associated with using digital technology for FP. However, midwives perceive that being well-educated about FP methods through technology may cause the woman a lot of problems with her husband, as she will use the information to justify her personal decisions, even if they are thoughtless or inconsiderate (eg, abortion).

#### Internet Access and Health Literacy

According to clients and midwives, most women have smartphones and internet service, and they use social media platforms all the time which makes it easier and faster for them to obtain family information; a participant explained, “we all have smart phones and access to the internet … we can ask questions online and get answers.” Midwives perceive that women are curious in nature and they tend to ask questions and answer them, and they like to get medical consultations through the websites. For example, a midwife explained, “women tend to believe what technology tells them; when one asks a question through Facebook groups, a hundred reply with completely different answers. She then comes to the center telling me that I asked and got the answers myself ....” A midwife explained that, “Mobile phones are available for all people, and women living here like to know about the FP methods. In most cases, internet services are available; even if the woman’s financial situation is not that good, she usually has a mobile phone and an internet connection.” However, as one of the concerns, midwives and some women believe that the technological support may be limited due to technical errors or constant internet disconnection. A participant explained, “I worry that the internet cuts off during the time I need to have answers regarding family planning.” Besides, a midwife at a rural health care center mentioned that technology may not be effective in the less developed regions. As people are still strict against using mobile devices at home, women may still not know how to use mobile technologies due to their low educational level or the impact of the surrounding society. It is also possible that some women may even be unable to afford owning a mobile phone. A midwife stated, “Some regions are far from the services, and they are still strict somehow.”

### Ideal App or Website Characteristics

The Jordanian and Syrian women and midwives described the ideal characteristics of a potential digital health app for FP. The needed and desired features mainly included visualizations and videos, being easy to understand, having a log-in feature (password protected) to maintain privacy, and free of charge accessibility.

#### Ease of Understanding and Visualizations

Most midwives thought that using digital health solutions for FP services will be highly and widely accepted by women under certain conditions. One major factor to take into consideration depends on the way of presenting and explaining information; a midwife explained that it will be more effective to use a simple language to be understandable by women of all educational and ideological levels. Most midwives reported that “video” is more effective than text as they believe some women are illiterate and they find videos catchier and more memorable. Some of the Jordanian and Syrian clients believed that information provided through videos is easier to understand and remember, especially for illiterate women, whereas others do not have issues with reading due to their high level of education. As we mentioned above, when talking about eHealth literacy, the level of education can be a barrier to interventions delivered through digital technology, especially among Syrian women, as, on average, their educational level is lower than that of Jordanian women, as was stated by a Syrian participant “usually in Syria, girls get off married early before they complete their 8^th^ grade,” so they drop-out from school at an early age. Hence, it is important to make sure that low literacy is considered. In fact, one client mentioned, “The website of mobile application should be presented with lots of visualization and videos, which will be more effective to understand and apply.” Another woman said, “I prefer watching a video because I am a visually oriented person.” However, some clients thought it would be a good idea to provide the information as both text and video to fit everyone’s taste. Some interviewees stated that videos are easier to use as they can pause or rewatch them when they are free, as noted by another participant, “I can watch the video any time I want and anywhere I am at.” A midwife also said, “women can watch the video in their free time or even listen to it while doing other tasks at home.” Some midwives added that women can show the videos to their husband or mother-in-law.

#### Privacy Concerns

When Jordanian and Syrian women were asked about the level of acceptance of digital technology for them and their husbands, all participants confirmed their acceptance. In fact, they stated that their husbands will not mind them accessing information online, if it is safe information, and no one can find out. This was noted by a participant: “my husband will make sure that no one except me and him know about using the mobile application.”

Some clients preferred using password-protected platforms to protect their privacy and prevent their children (especially young daughters) opening the app and reading information that is not appropriate for their age. A participant said, “If there will be videos explaining – how to use FP methods – then they prefer to have a login account.” Another participant explained, “I need to know that the application is secured by having a login account, so my daughter does not open it and read.”

#### Free Access

Many midwives believed that it is fundamental that many clients accept and utilize such services so that they become cost-effective. Apps and websites need to be free of charge so that anyone can access them, as some midwives lamented the fact that some clients would not pay to get FP-related services due to financial limitations.

## Discussion

### Principal Findings

This study provides a significant contribution to knowledge as it is the first to explore the perceptions of women from refugee and host communities and midwives in Jordan toward the potential use of digital technology for enhancing FP. Overall, the response of the Syrian and Jordanian women and midwives was positive. According to most of the participating women in our study, it would be ideal if FP was discussed and provided at every level of digital technology, especially through mobile apps and websites (or web applications).

### Advantages of Digital Technology

The study participants perceived that digital technology may serve as means to increase awareness and knowledge related to the different FP methods and their physical and mental side effects. In this regard, several previous studies have supported the use of digital technology in FP. For example, a study conducted by Greenleaf et al [38] showed that women who own a mobile phone were more likely to use modern FP methods compared with women who do not own one. Another study conducted in Bangladesh aimed to evaluate the effect of digital health package on FP knowledge and behavior through providing offline digital health training [39]. The findings showed improvement among women who received digital health package on FP regarding the choice of contraception, contraception method side effect, and management of side effects as compared with those without exposure at all. It was also found that prevalence of modern FP methods among women who received a digital health package on FP was high compared with those without exposure at all [39].

Another major advantage of digital technology is that it can aid the decision-making process and empower women. Almost all interviewed midwives and women (clients) agreed that digital and mobile technology could enable women and empower them to make informed decisions and can teach them how to make joint decisions with their husbands by following specific communication and bargaining skills and strategies. Several studies investigated the effect of digital and mobile technologies for FP on women empowerment and their influence on the decision-making process. A clustered randomized control trial conducted in Nigeria assessed the efficacy of the digital health tool ‘Smart Client’ on ideational and behavioral variables related to family [23]. The study researchers have found that the intervention group, which received regular mobile phone calls, showed improvements in the confidence level of women while discussing FP with a health care provider, and that women in this group tend to use modern FP methods more than those in the control group [23].

### Concerns About Digital Technology for FP

When we asked the study participants whether the mobile technology would replace counseling services provided by midwives, the majority disagreed, but mentioned that it can be developed in conjunction with the available services provided at medical health centers. A study in Kenya indicated that mobile health (mHealth) alone was unable to improve contraception knowledge and use [40]. Nonetheless, previous studies concluded that appropriate use of the mobile technology needs biomedical screening and counseling, thus empowering selection of FP methods based on lifestyle and priorities of women [41].

Women are increasingly fascinated by using science-based mobile technology in contraception and family practices. However, most of the available mobile apps support natural FP methods [41]. A previous study aimed to evaluate smartphone apps that are designed to help users of FP methods to prevent unintended pregnancy [42]. Of the 218 apps identified in that study, 12 scored 50 out of 90 points on features and 15 out of 21 points on contraception and pregnancy prevention best practice. Moreover, 41% of the apps did not contain any information about modern FP methods, and 21% contained information about 1 method only, with only half of the apps that briefed about modern contraception methods providing instructions for using FP methods [42]. Hence, digital health interventions should be aimed at modifications in behavior and shifts to new practices such as moving away from paper-based systems to digital methods [1]. Recently, the WHO has issued a guideline that provides 10 evidence-based recommendations on digital health interventions focusing on factors that influence the feasibility and acceptability of implementing digital health technologies, especially in low-resource settings, and taking into account gender, equity, and human rights issues [1]. Therefore, health care providers are encouraged to follow this useful WHO guideline when developing or implementing digital health interventions. At the same time, we believe that researchers should carefully design interventions taking into account the different levels and domains of literacy that pertain to the eHealth literacy model [37]. In our study, both clients and providers mentioned the need to provide content in a visual format, catering for participants with low literacy levels, and to make it available in an easy-to-use format. Curating content and making sure the information is accurate and correct are also fundamental elements of digital interventions for FP, which can avoid the diffusion of misinformation and disinformation (myths and wrong beliefs) about FP [43].

### Ideal Apps or Websites

Based on the opinions shared by our study participants, mobile apps for FP should complement—not substitute—the information and services available, linking clients to providers. The information should be accurate, easy to understand, and verified by professionals. The apps or web services should be easy to use, based on videos or a combination of visual and textual information, accessible without the internet, and password protected. These services should be free of charge.

### Strengths and Limitations

This qualitative study has several strengths. On one hand, the narratives included the perceptions of women and midwives to ensure triangulation of data and maximize the credibility and trustworthiness of the results. On the other hand, the researchers who conducted the data collection and analysis had vast experience in qualitative research methods. As for the limitations, as with most qualitative research designs, the study findings cannot be generalized to a wider population especially because we only included 2 governorates in Jordan. However, the study findings offer in-depth insights into personal and contextual factors affecting digital technology use for enhancing FP in Jordan.

### Conclusions

As perceived by midwives and women, incorporating digital technology in FP services can be feasible, cost-effective, well accepted, and potentially beneficial in increasing woman’s awareness regarding the FP methods and their side effect. It also may improve shared decision-making process among Jordanian couples. Therefore, digital technologies are recommended to address the challenges faced in health system and to improve both the coverage and quality of FP services and practices.

## Abbreviations

FGDs: focus group discussions
FP: family planning
GDHI: Global Digital Health Index
IDRC: International Development Research Center
IRC: International Rescue Committee
LMICs: low- and middle-income countries
mHealth: mobile Health
RQDA: Qualitative Data Analysis in R
UNHCR: United Nations High Commissioner for Refugees
WHO: World Health Organization
